# CardioVar: a machine learning framework for pathogenicity prediction of cardiomyopathy genetic variants

**DOI:** 10.1093/bioadv/vbag135

**Published:** 2026-05-12

**Authors:** Nabras Al-Mahrami, Aaisha Albalushi, Fahad Al Hattali, Mashael Al Balushi, Bushra Al Shamsi, Musallam Al-Oraimi, Tuqa Al Lawati, Mohamed Al-Rawahi, Nadia Alhashmi, Ahmed Al-Amri

**Affiliations:** Medical Laboratory Sciences Program, Oman College of Health Sciences, Muscat, PO 112 , Oman; National Genetic Center, Royal Hospital, Muscat, PO 111, Oman; National Genetic Center, Royal Hospital, Muscat, PO 111, Oman; National Genetic Center, Royal Hospital, Muscat, PO 111, Oman; National Genetic Center, Royal Hospital, Muscat, PO 111, Oman; National Genetic Center, Royal Hospital, Muscat, PO 111, Oman; National Heart Center, Royal Hospital, Muscat, PO 111, Oman; National Heart Center, Royal Hospital, Muscat, PO 111, Oman; National Genetic Center, Royal Hospital, Muscat, PO 111, Oman; National Genetic Center, Royal Hospital, Muscat, PO 111, Oman

## Abstract

**Motivation:**

CardioVar is a machine learning framework developed to predict the pathogenicity of cardiomyopathy-associated genetic variants, providing a rapid and disease-focused classification score to support variant interpretation in cardiogenetic workflows.

**Results:**

This approach supports variant interpretation within cardiac-related genes and may complement existing pan-disease models within a cardiomyopathy-specific context. In prospective case study settings, CardioVar demonstrated an 85% concordance with conventional tertiary analysis results.

**Availability and implementation:**

https://github.com/nibrasissa/CardioVar

## 1 Introduction

Cardiomyopathies represent a heterogeneous group of diseases affecting the myocardial structure and function, with different non-genetic and genetic etiologies (Kraus *et al.* 2024). These disorders can be broadly classified into Hypertrophic Cardiomyopathy (HCM), Dilated Cardiomyopathy (DCM), Arrhythmogenic Cardiomyopathy (AC), and Restrictive Cardiomyopathy (RCM), each characterized by unique histopathological and clinical features (Jin *et al.* 2025). While environmental factors such as hypertension, viral infections, and lifestyle contribute to disease expression, a substantial fraction of cardiomyopathy cases arise from germline genetic variants. To date, >100 genes have been implicated in inherited cardiomyopathies, encoding sarcomere proteins (e.g. MYH7 and MYBPC3), cytoskeletal elements (e.g. TTN), desmosome components (e.g. PKP2), and ion channels (e.g. SCN5A) (Ciarambino *et al.* 2021). The clinical interpretation of cardiogenetic variants is one of the major challenges to the practice of genomic medicine, due to several factors, including phenotypic heterogeneity, high consanguinity rates, gene penetrance, and multiple modes of inheritance within the same gene (Pepin *et al.* 2016, Brlek *et al.* 2024). The American College of Medical Genetics and Genomics and the Association for Molecular Pathology (ACMG-AMP) provide guidelines on the use of various evidence to determine the pathogenicity status of variants. A large proportion of variants encountered in practice remain “variants of uncertain significance” (VUS), indicating the need to improve evidence utilization practices (Richards *et al.* 2015, Chen and Guo 2020, Wain *et al.* 2020). Public databases, such as the phenotype database [e.g. Online Mendelian Inheritance in Man (OMIM)], population database (e.g. gnomAD), and *in-silico* prediction tools (e.g. CADD), provide powerful resources but differ in scope, calibration, and interpretability (Leong *et al.* 2015). Providing a framework for integrating different resources into the cardiomyopathy context might represent more robust techniques for variant classification.

The recent discipline of machine learning (ML) has been successfully used in different aspects of genomics data integration (Zhu *et al.* 2025). It focuses on the creation and deployment of computer algorithms that progress with inputs, optimize error rates, and predict outputs. These algorithms are effective enough to cope with enormous datasets characterized by incoherence, noise, and high dimensionality, with few indications about the underlying data distribution (Libbrecht and Noble 2015). Currently, many existing tools are working by integrating diverse annotation information with different ML algorithms to predict variant pathogenicity status, such as Learning from Evidence to Assess Pathogenicity (LEAP) (Lai *et al.* 2020), which has been developed in a clinical laboratory setting by using 5398 missense variants in 30 cardiovascular genes and 4226 missense variants across 24 hereditary-cancer genes. Each variant was annotated by a wide spectrum of information, including functional and conservation scores, splice predictions, variant location/protein domains, population frequencies, and selected individual-level signals. Then, logistic regression and random forest models were used for training and testing. LEAP demonstrated high performance in 10-fold cross-validation, with an AUROC of ∼98% for cancer and cardiovascular cohorts. Another computational approach of combining several *in-silico* predictors, including both deleteriousness and conservation scores, showed a wide variability in concordance (Ghosh *et al.* 2017). Moreover, CardioClassifier (Whiffin *et al.* 2018) is a semi-automated tool for inherited cardiac conditions that applies disease- and gene-specific ACMG/AMP rules across 40 cardiac genes. Benchmarked on 57 curated variants, it achieved 87.3% rule concordance and identified more than double the clinically actionable variants compared to a generic tool, with a diagnostic yield of 33.7% in 327 cardiomyopathy cases. While effective, its rule-based design and focused gene panel highlight the potential for machine learning approaches to extend variant interpretation across broader gene panels. While existing tools are available for all sorts of variants, a dedicated ML for cardiomyopathy genetic variants has the potential to contextualize the pathogenicity status and highlight the drawbacks of existing ML tools. According to several studies, the predictive performance varies according to training and testing datasets, feature selection, and the specific ML algorithms (Gönen and Alpaydın 2011, Saligan *et al.* 2014).

Here, we present the CardioVar tool as an end-to-end ML framework for predicting the pathogenicity of cardiogenic variants, intending to contextualize pathogenicity prediction within a cardiomyopathy-specific gene panel. Whether this disease-focused approach captures patterns not optimized in general-purpose classifiers remains a hypothesis that warrants future comparative benchmarking.

## 2 Methods

### 2.1 Data source

We obtained all reported variants from ClinVar (downloaded on 26 May 2025) (Landrum *et al.* 2018). ClinVar is a public database that contains human genetic variants with their pathogenicity classification, submitted by diagnostic and research communities and maintained by the National Center for Biotechnology Information (NCBI). To focus on inherited cardiac disease, we restricted analyses to a curated cardiogenetic panel comprising 250 genes implicated in cardiomyopathies and inherited arrhythmia syndromes. The gene list (Table 1, available as supplementary data at *Bioinformatics Advances* online) was used to filter ClinVar entries by gene symbol before annotation. Analyses were limited to single-nucleotide variants (SNVs) and short indels mapped to the genome build GRCh38/hg38. Clinical significance was harmonized from ClinVar’s assertion field (CLNSIG). Variants annotated as pathogenic or likely pathogenic were assigned label 1, and benign or likely benign were assigned label 0. VUS and any conflicting interpretations or missing values were excluded from modeling to minimize label noise. All variants were functionally annotated using ANNOVAR (release version 2020060, 29 July 2025) (Wang *et al.* 2010) with standard gene-based, region-based, and filter-based protocols. At annotation time, 114 annotation fields were retained, including population frequency databases, *in-silico* predictors (dbNSFP bundle), and consequence/impact (Table 2, available as supplementary data at *Bioinformatics Advances* online).

**Table 1:** Comparative performance metrics of five ML models using the he hold-out test set.

Model	Precision	Recall	Specificity	F1	Accuracy
**XGBoost**	0.98	0.98	1.00	0.98	0.99
**Gradient boosting**	0.98	0.97	1.00	0.97	0.98
**Random Forest**	0.96	0.98	0.99	0.97	0.98
**SVM**	0.96	0.97	0.99	0.96	0.96
**Logistic regression**	0.94	0.97	0.99	0.96	0.91

### 2.2 Dataset and feature preparation

During annotation, some variants have missing or encoded as blanks, dots, or nulls in the dataset file. We standardized missingness encodings to NA and applied minimal validity filters to remove records that would bias downstream analyses. Duplicate variants with the (same CHR: POS: REF: ALT) collapsed, retaining one record per unique allele. Missing values in retained predictors were handled during model preprocessing by mean imputation for numeric and most-frequent imputation for categorical features. Additionally, low-cardinality fields (with ≤30 unique values) were retained and one-hot encoded, while free-text/high-cardinality columns were removed. The ClinVar clinical significance field (CLNSIG) was used as the training label and was not included as an input feature. Also, all identifiers (coordinates, IDs, gene symbols) and label-derived fields from ClinVar were explicitly excluded from the feature matrix prior to model training to prevent information leakage. The final dataset description is summarized in Section 3.

### 2.3 Models training and testing

The data were divided with a fixed, stratified hold-out strategy (seed = 42) to ensure class balance and prevent optimistic bias. First, 20% of the records were held out as an unreserved test set that was not used for any model’s training, feature selection, or hyperparameter optimization. The remaining 80% was divided further into internal training (64%) and internal validation (16%) subsets. All the transitions were trained on the training subset only to avoid information leakage. We compared regularized logistic regression, random forest, gradient boosting, XGBoost, and RBF kernel SVM. These five algorithms were selected to cover a broad spectrum of machine learning approaches, from a simple linear model (logistic regression) to more complex ensemble and kernel-based methods. Hyperparameters were tuned for each model class using RandomizedSearchCV with StratifiedKFold (*k* = 5) on the train subset. The objective of the tuning was to maximize average precision (AUPRC), which was selected a priori to capture the class imbalance characteristic of clinical variant interpretation. Strength of regularization of search spaces (C for logistic regression/SVM), width of kernel (gamma), number of trees and depth, learning rate, subsampling, and minimum leaf sizes 9 (Table 3, available as supplementary data at *Bioinformatics Advances* online). Activated upon use, feature reduction used a two-step procedure on the training subset only: (i) mutual information pre-selection retained top-*K* candidates (default *K* = 300) and (ii) greedy forward selection incrementally added features that maximally improved cross-validated AUPRC with the help of a light logistic-regression proxy, with early stopping (patience = 3) and max 30 features. If feature selection identified a beneficial subset, models were trained on that subset; otherwise, all available features were retained for training. After tuning, the best estimator of each family (with the highest mean CV AUPRC) was evaluated on the internal validation subset for diagnostic testing and subsequently re-estimated on the combined training data (internal training + internal validation) with selected preprocessing and selected features kept constant. This held model was evaluated once on the held-out 20% test set. The data preprocessing, feature engineering, and modeling pipeline were implemented using Python v3.10 (see Section 3).

### 2.4 Model performance

Primary performance was AUPRC on the holdout set. Secondary performance measures were AUROC and Brier score. For reporting decision-level behavior, we computed confusion matrices and calculated precision, recall (sensitivity), specificity, F1, and accuracy at two thresholds: the default 0.50 and the F1-optimal threshold from holdout predictions. For interpretability, SHAP values were computed on a predefined sample of the holdout set using model-appropriate explainers (tree or kernel). We summarize global importance with beeswarm and mean bar plots and provide representative local explanations. Low-dimensional visualization of the holdout data (post-preprocessing) was obtained using t-SNE with fixed parameters for comparability across models.

### 2.5 Omani cardiomyopathy cohort

Between 2024 and 2025, we enrolled 60 unrelated families with a clinical diagnosis of cardiomyopathy and a strong positive family history seen at the Royal Hospital, Muscat, Oman, as part of a cardiomyopathy AI project funded by the government of Oman. Ethical approval was obtained from the Ministry of Health, Muscat, Oman (MoH/CSR/23/26793). All participants signed the enrollment consent form.

### 2.6 Sample collection, DNA extraction, and sequencing

Blood samples were collected from all participants through antecubital fossa venipuncture using EDTA vacutainer tubes. DNA extraction was performed using the Hamilton robotic system, which utilizes magnetic bead-based technology to ensure high-purity DNA. This process requires 1 ml of blood and yields ∼200 µl of purified DNA with at least 50 ng/µl concentration for downstream applications. The DNA from a single affected case in each family proceeds into Whole Exome Sequencing (WES). The library preparation was performed using TWIST reagents, and sequencing was carried out on a DNBSEQ-T7 (MGI) instrument with PE150-FCL cartridges. Each sample targeted ∼10 Gbp of data, achieving an average target coverage of 100-120×.

### 2.7 Bioinformatics analysis

Reads were aligned to GRCh38/hg38 with BWA-MEM; marking of duplicates, base quality recalibration, and indel realignment followed GATK Best Practices (McKenna *et al.* 2010). Single-nucleotide variants (SNV) and indels were called with HaplotypeCaller, and Standard QC thresholds were applied: DP ≥ 10, GQ ≥ 20, and strand bias filters. Each case was interpreted double-blind by two certified molecular geneticists and one bioinformatician using OmnomicsNGS (Euformatics, Finland) and Exomiser (Pengelly *et al.* 2017) for tertiary analysis. Variant call files (VCFs) were imported into each tool, and the workflow comprised (i) application of quality and frequency filters (gnomAD), (ii) consequence-based prioritization (protein-altering and splice-relevant events), and (iii) aggregation of *in-silico* predictions and ClinVar evidence, all matched to GRCh38/hg38 coordinates. Discrepancies between the two blinded classifications were resolved in a consensus meeting (geneticists + bioinformatician), with the final classification and rationale recorded in the case log.

### 2.8 CardioVar application to cardiomyopathy cases

For each proband, we re-annotated candidate variants identically to those in the training set, then applied the best frozen model pipeline (preprocessing and feature set fixed; no retraining). Model outputs consisted of probabilities of pathogenicity per variant, along with SHAP attributions (Yao *et al.* 2023).

### 2.9 CardioVar web application

We provided CardioVar as a browser-accessible web application that serves variant-level pathogenicity predictions from the frozen best model developed in this study. The app accepts pre-annotated variants (SNVs and short indels mapped to GRCh38/hg38) and returns a calibrated probability of pathogenicity.

## 3 Results

**Figure 1:**
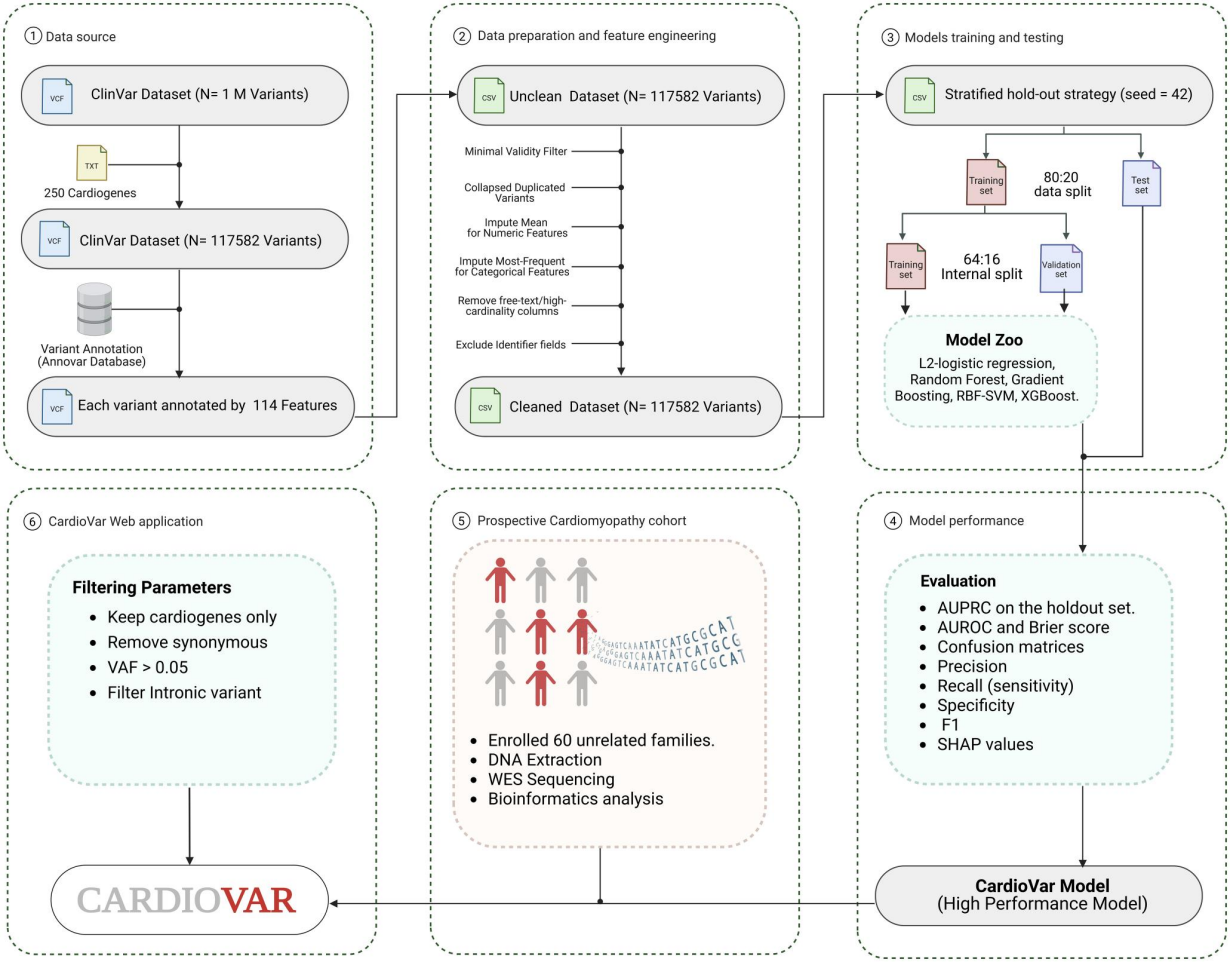
CardioVar development and evolution workflow. Genetic variants were retrieved, annotated, data preparation and feature selection and modeled with several ML algorithms, evaluated using various performance matrices, and then validated by a cardiomyopathy cohort.

We initially set up the CardioVar farmwork by performing a series of processes as illustrated in (Fig. 1). In step 1, the ClinVar database (https://www.ncbi.nlm.nih.gov/clinvar/) is used as a source of variants associated with cardiomyopathies. The retrieved dataset has been filtered using the cardiomyopathy comprehensive genes panel, which contains 250 reported genes. Accordingly, 117 582 cardio gene variants were annotated using 114 features with ANNOVAR. In step 2, the data preprocessing reveals [Pathogenic/Likely Pathogenic = 16 725 (14.22%), Benign/Likely Benign = 100 857 (85.78%)] for downstream modeling. In steps 3 and 4, we implemented a stratified hold-out strategy with seed = 42 to provide a balanced distribution between training and test sets. Several ML models were trained for comparison as a model zoo, aiming to provide the best-performing model for CardioVar deployment. In step 5, a prospective recruitment of 60 patients with cardiomyopathy was used as further validation in a clinical setting. All the cases were analyzed using a conventional variant filtering process. In step 6, we streamlined CardioVar as a web tool to facilitate its utilization. CardioVar is provided as a browser-accessible web application and is publicly available at https://github.com/nibrasissa/CardioVar, with installation instructions provided for both Windows and macOS/Linux environments. Users may upload pre-annotated variant files in CSV format and receive calibrated pathogenicity probability scores as downloadable output.

### 3.1 Dataset and feature preparation

**Figure 2:**
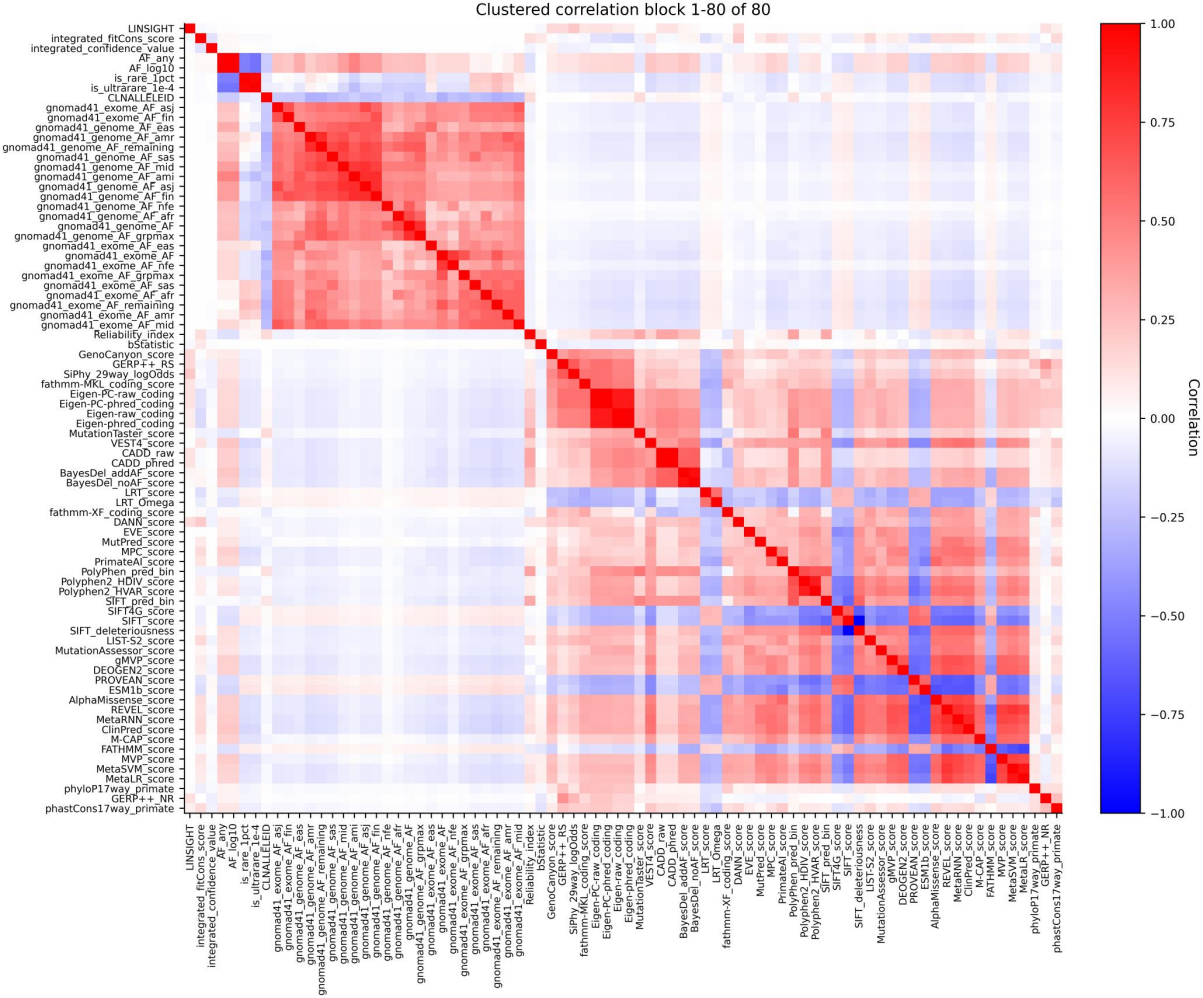
Correlation structure of engineered predictors. Spearman correlations were computed among all numeric features in the ClinVar cardiogene dataset after preprocessing (*N* = 117 582 variants).

The retrieving process revealed 117 582 cardiomyopathy variants annotated with 114 features. The initial dataset contained multiple transcript entries for each genetic variant. To ensure data integrity and avoid redundancy, we removed the variant duplicates by selecting canonical transcript only. This approach preserves the total number of variants. Also, the preparation process excluded 34 features. Eighty features related to population frequency databases, *in-silico* predictors (dbNSFP bundle), and consequence/impact were suitable for the downstream process. We examined the correlation structure of features using the Spearman correlation matrix as illustrated in Fig. 2. The population allele frequency metrics demonstrated a dense block of positive correlations with each other (*r* > 0.95). The *in-silico* predictors form clusters together, indicating shared predictive information. In contrast, a few of the conservation features, such as phyloP and phastCons, have moderate correlations to pathogenicity predictors. These patterns highlighted the potential role of feature selection.

### 3.2 Model’s performance

**Figure 3:**
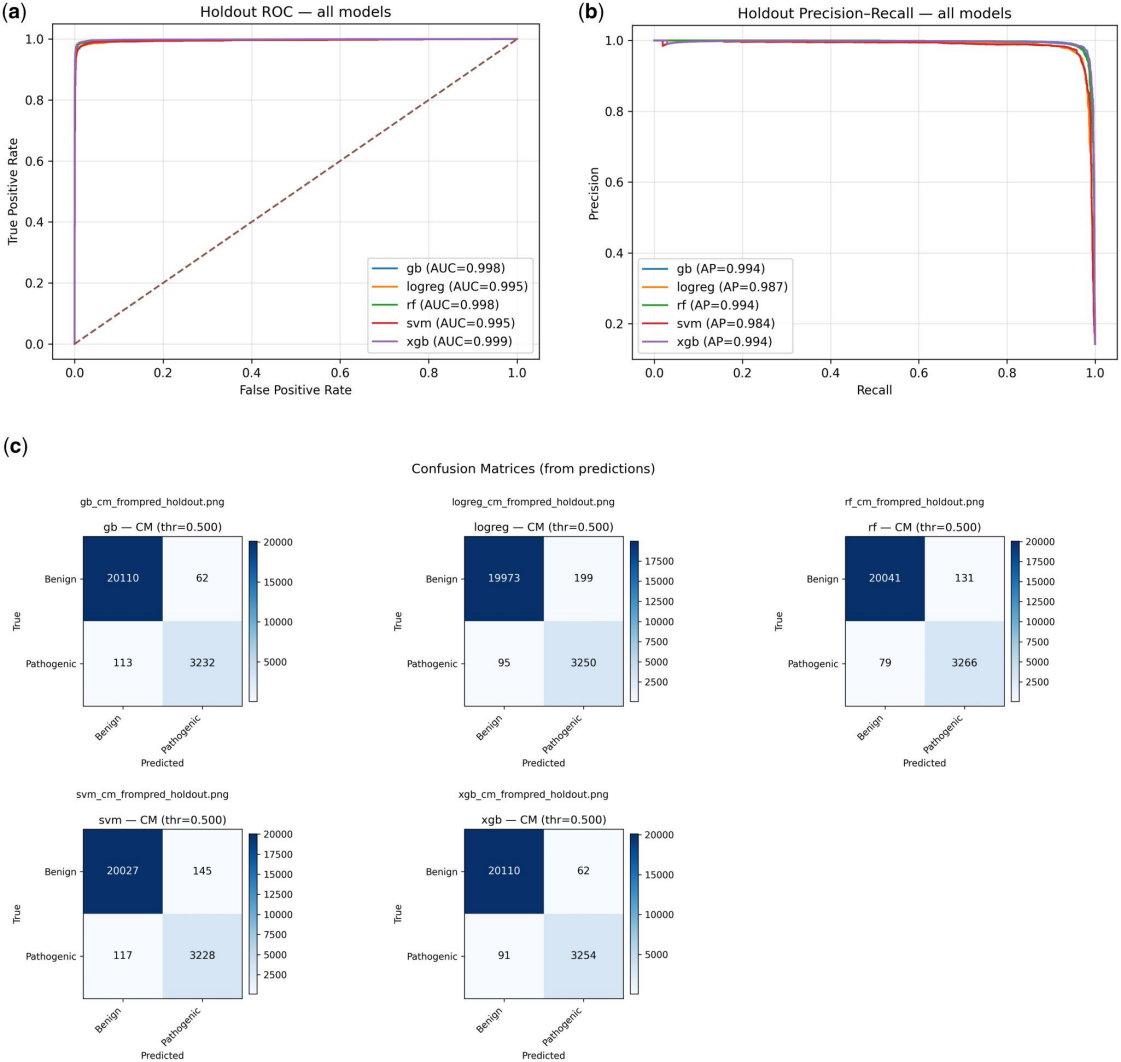
Holdout performance of five classifiers for variant pathogenicity prediction. (a) Receiver operating characteristic (ROC) curves for gradient boosting (gb), logistic regression (logreg), random forest (rf), RBF-SVM (svm), and XGBoost (xgb) evaluated on the 20% holdout set. (b) Precision–recall (PR) curves on the same holdout set, optimized for class imbalance. (c) Confusion matrices at the default probability threshold of 0.50 for each model.

We demonstrated the ability of five machine learning models, including XGBoost, Gradient Boosting, Random Forest, Support Vector Machine (SVM), and Logistic Regression to classify variants into pathogenicity status based on a hold-out test set. Assessing the discriminative performance of each model using AUPRC and AUROC (Fig. 3a) and Precision–recall (PR) analysis (Fig. 3b) demonstrates a strong separation between the two classes, with values ranging from 0.96 to 0.98. The confusion matrix analysis (Fig. 3c) provides deeper insight into misclassification. Across all the models, the XGBoost model misclassifies 62 false positives and 91 false negatives out of over 23 500 variants. The performance accuracy approaches 0.99 in all models. However, the XGBoost showed a balance between precision (0.98) and recall (0.97), providing the highest F1-score. The remaining models performed slightly less optimally, as shown in Table 1. According to the SHAP feature importance, the top-ranked features were meta-predictors (MetaRNN, gMVP, and MVP), which combine multiple *in-silico* tools into a single pathogenicity score. Population allele frequency (gnomAD) ranked lower, suggesting that functional impact scores are more informative than frequency filters within a cardiomyopathy-specific gene panel. This is expected, as pathogenic cardiomyopathy variants can occur at low but detectable frequencies in the general population, unlike truly rare variants in pan-disease models (Appendix I, available as supplementary data at *Bioinformatics Advances* online).

### 3.3 Application of CardioVar using prospective cases

The application of CardioVar was assessed using 60 cases from unrelated families. These cases were clinically evaluated to represent an inherited form of cardiomyopathy from different age groups. All the cases were successfully sequenced and passed primary and secondary bioinformatics analysis pipelines. These steps provide millions of variants to be assessed by CardioVar and the conventional filtering pipeline. Of the 60 recruited cases, 51 (85%) showed concordant classification of the candidate causative variant by both the conventional filtering pipeline and CardioVar model. Only nine cases (15%) showed a discordant finding, indicating the current limitation of the selected model. These case studies provide early proof of concept for the model’s applicability in real clinical settings and support the potential utility of disease-specific machine learning for cardiomyopathy variant interpretation.

In this research project, we developed a meta-predictor called the CardioVar model for classifying cardiomyopathy genetic variants from a massively annotated file. The CardioVar development workflow utilizing the publicly reported variants. The retrieved dataset was composed of more benign variants than pathogenic; therefore, the merging of pathogenic and likely pathogenic variants under the label (pathogenic) and benign and likely benign under the label (benign) and likely pathogenic under the label (pathogenic) resulted in structuring the datasets and helped boost classifier performance. This binary framework maximizes label reliability; however, CardioVar was not trained to differentiate VUS from either class, and in clinical application, scores assigned to VUS should be interpreted as a continuous probability estimate to support manual expert review. The evaluation of different ML models suggests that various classifiers can accurately classify variants with no missing feature values. However, the real clinical setting demonstrated a different level of accuracy;

Therefore, further validation in a clinical setting according to the regulations of the World Health Organization’s guidance on the Ethics and Governance of AI for Health is required (World Health Organization 2021).

Therefore, we utilized a high-quality WES dataset of cardiomyopathy patients as case studies to illustrate the model’s functionality and validation method, starting from an annotated VCF file. The CardioVar demonstrated 85% matching results with conventional tertiary analysis in a real clinical setting. This represents the influence of pre-processing on producing a clean dataset, while clinical setting testing provides further insight for optimization.

Narrowing down the analysis workflow via the ML application according to the disease of interest demonstrated more robust processing (Park *et al.* 2021). In previous studies, the ML application has been adopted for wider applications rather than disease-specific ML for variant classification. For example, Rare Exome Variant Ensemble Learner (REVEL), which is an Ensemble model combining outputs from multiple tools (e.g. SIFT, PolyPhen-2, and MutationTaster). The performance of REVEL showed an AUC of 0.91 in an independent test set of 65 000 exome variants (Ioannidis *et al.* 2016). Unlike general-purpose classifiers, CardioVar was trained exclusively on a 250-gene cardiomyopathy and inherited arrhythmia panel, allowing disease-focused variant interpretation. It was prospectively applied to a clinical cohort from Oman, a non-Western, high-consanguinity population underrepresented in most existing pathogenicity tools. The framework covers a full workflow from variant annotation to a web-accessible probability score, and includes feature importance analysis to support result interpretation. We present CardioVar as a complementary tool to support variant interpretation in cardiogenetic workflows, rather than a replacement for expert clinical analysis.

CardioVar demonstrates that disease-specific machine learning can support variant interpretation for cardiomyopathies, showing reasonable concordance with expert analysis in a prospective clinical cohort. Limitations include dependency on the diversity of the training set and the need for ongoing model retraining as new variant data emerge. Additionally, the possibility of overfitting cannot be fully excluded, as the model was trained on curated ClinVar variants that may not capture the full complexity of real-world clinical cases. Future work suggests the utilization of cross‐institutional datasets and evaluation of CardioVar against REVEL, LEAP, and CardioClassifier on a common held-out benchmark to establish its comparative utility.

## Supplementary Material

vbag135_Supplementary_Data
