# Supplementary material for: CardioVar: a machine learning framework for pathogenicity prediction of cardiomyopathy genetic variants
Source: Bioinform Adv. 2026 May 12;6(1):vbag135. doi: 10.1093/bioadv/vbag135 (PMC13215092; doi:10.1093/bioadv/vbag135)
Supplement: vbag135_Supplementary_Data [file vbag135_supplementary_data.zip › Appendix_I.docx]

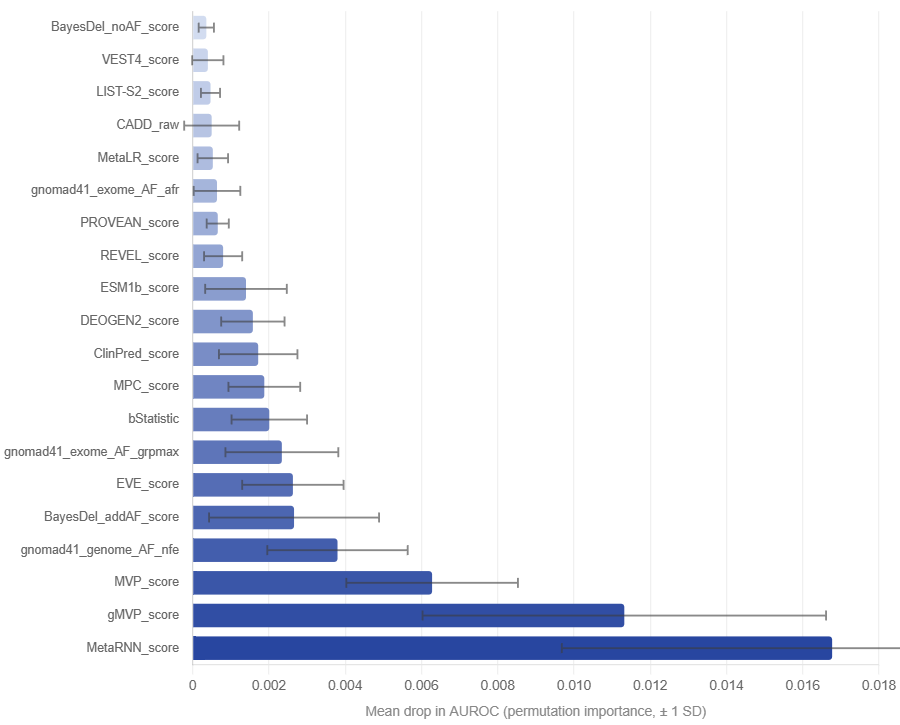


Figure 1.Mean permutation importance (top 20 features). The features importance analysis identified MetaRNN_score as the most influential feature (importance = 0.017 ± 0.007), contributing substantially more than all other features, followed by gMVP_score (0.011 ± 0.005) and MVP_score (0.006 ± 0.002). Notably, the top three features are all ensemble meta-predictors that integrate multiple in-silico tools into a single pathogenicity estimate, reflecting their strong discriminative power within the cardiomyopathy variant context. Population allele frequency metrics gnomad41_genome_AF_nfe (0.004 ± 0.002) and gnomad41_exome_AF_grpmax (0.002 ± 0.001) contributed meaningfully but ranked lower than functional scores, suggesting that within a disease-specific gene panel, functional impact carries greater discriminative weight than frequency-based filters alone. This pattern is biologically consistent with cardiomyopathy genetics, where pathogenic variants can occur at low but non-negligible population frequencies due to variable penetrance and founder effects. EVE_score (0.003 ± 0.001), an evolutionary model-based predictor derived from protein sequence variation, ranked sixth, highlighting the added value of protein language model features beyond traditional conservation scores. The remaining features, including CADD_raw, REVEL_score, and BayesDel scores, contributed modest but non-zero importance, indicating that no single feature fully captures pathogenicity and that the ensemble approach of CardioVar benefits from integrating a diverse feature set.
